# The Effects of Load Carriage and Physical Fatigue on Cognitive Performance

**DOI:** 10.1371/journal.pone.0130817

**Published:** 2015-07-08

**Authors:** Marianna D. Eddy, Leif Hasselquist, Grace Giles, Jacqueline F. Hayes, Jessica Howe, Jennifer Rourke, Megan Coyne, Meghan O’Donovan, Jessica Batty, Tad T. Brunyé, Caroline R. Mahoney

**Affiliations:** 1 U.S. Army Natick Soldier Research, Development, and Engineering Center, Natick, Massachusetts, United States of America; 2 Department of Psychology, Tufts University, Medford, Massachusetts, United States of America; Purdue University, UNITED STATES

## Abstract

In the current study, ten participants walked for two hours while carrying no load or a 40 kg load. During the second hour, treadmill grade was manipulated between a constant downhill or changing between flat, uphill, and downhill grades. Throughout the prolonged walk, participants performed two cognitive tasks, an auditory go no/go task and a visual target detection task. The main findings were that the number of false alarms increased over time in the loaded condition relative to the unloaded condition on the go no/go auditory task. There were also shifts in response criterion towards responding yes and decreased sensitivity in responding in the loaded condition compared to the unloaded condition. In the visual target detection there were no reliable effects of load carriage in the overall analysis however, there were slower reaction times in the loaded compared to unloaded condition during the second hour.

## Introduction

Research examining the relationship between exercise and cognition typically focuses on the chronic, beneficial effects of exercise on cognition, especially in aging populations. In the case of acute exercise, there is evidence that cognitive aspects of performance are affected during heavy exertion [1]. In some occupations, such as the military, personnel are under extreme physical strain, often times carrying in excess of 45 kg for extended periods of time [2]. In military contexts, strain-induced decrements in cognitive performance can have potentially fatal consequences. However, there are few studies that quantify cognitive performance *during* prolonged exertion. Typically studies examine the pre and post effects of exercise on cognitive performance or performance changes during relatively short bouts of exercise lasting for under an hour, for a review see [3]. The current study aimed to quantify cognitive performance during a two hour bout of acute exercise during which participants carried a heavy load (40 kg) compared to when they carried no load while walking over flat versus graded terrain.

While anecdotal evidence suggests long periods of intense exercise should lead to performance decrements, many studies examining the effects of acute exercise on cognition show small effects of exercise on cognition [3, 4]. However, in one meta-analysis examining acute, intermediate intensity exercise, i.e. 50–70% VO_2max_, differential effects of accuracy and reaction speed were found on working memory tasks. The meta-analysis revealed heterogeneous, but significant effect sizes for both response time and accuracy, but in opposite directions. Acute, intermediate intensity exercise speeded response time and impaired accuracy in the majority of studies, but not necessarily due to a speed-accuracy trade-off. Enhanced response time was theorized to stem from activation of the autonomic nervous system, resulting in increased catecholamine activity, including norepinephrine and dopamine. While the McMorris and colleagues [5] meta-analysis revealed effects of intermediate intensity exercise on cognition, in many other studies, the duration and intensity of exercise is insufficient to produce fatigue states that mirror the extreme conditions under which military personnel operate or more generally would lead to cognitive performance decrements in the general population. Because of the inconsistency in methodologies, not only in duration and intensity of exercise, but also when cognition is measure (pre/post is most common) there is limited generalizability of these findings to cognitive performance *during* prolonged exertion.

While the majority of studies examining cognition post exercise show small positive effects [3, 4], when cognitive performance is measured during exercise both positive and negative effects have been found. Meta-analysis results suggest that within the first 20 minutes of an exercise task, exercise effects are negative [3, 4]. This may especially be the case if exercise is high intensity or performed by individuals of lower fitness abilities and/or those not practiced at making decisions while exercising [6–9]. However, as the length of exercise bout increases past 20 minutes, exercise enhances general cognitive processing [10–12]. However, these studies typically do not examine performance past 1 hour. Among the few studies that have, simple response time became slower after 10 minutes and then faster after 40 minutes of a 90 minute run [13]. More complex tasks such as perceptual response and map recognition improved after the first hour of three hours of cycling, but declined after two hours [14]. Thus research on the time course of cognitive performance during longer duration exercise is relatively limited.

In addition to the duration of exercise, the types of cognitive tasks influenced by acute exercise are mixed and seem to interact with duration of exercise. Designs with shorter, less intense aerobic exercise interventions show either null or positive results on executive functioning [12, 15, 16], but designs with longer, more intense aerobic exercise interventions show decrements [9, 17]. Dissociations have also been observed between increased reaction times and decreased accuracy during working memory [5] with moderate intensity exercise improving response time in working memory tasks during exercise, but with decreased accuracy. Any decrements in executive functioning seem to disappear if tested following exercise, as executive functioning improves both immediately following exercise as well as after a delay following exercise [4].

Fitness level of participants has also been shown to influence results during exercise. Those with high levels of physical fitness were found to have positive effects of exercise, whereas those with moderate levels of physical fitness experienced null results, and those with low levels of physical fitness sustained decrements in performance [9]. Labelle et al.’s [9] findings support differential effects for high and low fit individuals. When cognitive testing occurred immediately following exercise, those characterized as both high and low in fitness level experienced improvements that were not documented in moderately fit individuals [9].

As for a mechanism underlying the patterns of performance during exercise, Dietrich [18] offers the transient hypofrontality hypothesis as well as the more recent reticular-activating hypofrontality (RAH) model [19]. According to these hypotheses/models, there is a decrease in frontal neural activity due to the demands of exercise. Specifically, because exercise recruits activity in motor pathways (e.g. primary and secondary motor cortices, basal ganglia, cerebellum) as well as sensory (e.g. primary sensory cortex) and autonomic pathways (e.g. hypothalamus), structures supporting higher-level cognitive processes, including the prefrontal cortex, are disengaged, whereas motor performance is enhanced. Alternative explanations for cognitive performance during exercise can be linked to activation of the autonomic nervous system, resulting in increased catecholamine activity, including norepinephrine and dopamine as explanations for enhanced reaction time while exercising [5].

Overall, the patterns of cognitive performance during exercise are variable and often contradictory. However, some factors, such as participant’s level of physical fitness, time when performance is measured, and intensity of exercise all seem to play an important role in determining whether or not improvements or decrements in performance are observed. The current study aimed to measure performance during exercise, focusing on two different types of tasks, one reliant on executive control and inhibition (auditory go/no-go) thought to involve brain structures down-regulated during acute exercise [18, 19], and another on visual vigilance (visual target detection task) thought to involve motor and sensory pathways in the brain.

During two hours of prolonged walking, we manipulated the intensity of exercise by having participants carry either 40 kg or no load at a constant treadmill speed of 1.35 m/s. For the purposes of this study, and based on our previous work, a +4% grade was used to investigate the onset of physical fatigue during a prolonged march. We operationally defined physical fatigue as exertion at or above 50% (±5%) of an individual’s peak rate of oxygen uptake ($\dot{V}{\text{O}}_{2\text{Peak}}$). To induce physical fatigue, the treadmill grade in this was set at +4% for the first 60 minutes of the march and then the participants walked for an additional 60 minutes on either a constant downhill grade of -8% or on varied grades of -8%, 0%, and +4%. The grade of the treadmill was manipulated during the second hour to examine physiological and cognitive performance recovery during downhill compared to variable grade walking. We expected the percentage of *VO*_*2* Peak_ would decrease during the second hour of walking at the downhill grade and parallel the changes in grade in the variable condition; however, we did not expect the same recovery in cognitive performance.

Few previous studies [13, 14, 20] have examined the time course of cognitive performance throughout prolonged exercise. On the basis of the hypofrontality hypothesis, we predicted that performance on the inhibition task (go/no-go) would be most sensitive to the manipulation of load as this task relies upon frontally dependent cognition, whereas, we expected performance on the visual vigilance task to remain mainly intact. However, most of the previous research has examined relatively short bouts of exercise and therefore, this pattern may not extend to longer duration bouts of acute exercise.

## Methods

### Participants

Ten male U.S. Army enlisted soldiers volunteered and completed this study (18–30 years old, *M*_*age*_ = 22.6, *SD*_*age*_ = 3.6 years). Data from an additional three participants was available for the visual target detection task, however for the sake of comparison, only participants who had data from both paradigms were included in the main analysis. The study took place in the Biomechanics Laboratory at the U.S. Army Natick Soldier Research, Development, and Engineering Center (NSRDEC, Natick MA). Height, weight, the load percentage of body weight (40 kg/participant weight), *VO*_2_ peak, two mile run time, and physical fitness test (PT) scores were collected from all participants (summarized in Table 1). This study was approved by the United States Army Research Institute of Environmental Medicine (USARIEM) Institutional Review Board. Participants gave written informed consent in accordance with approvals granted by the USARIEM Institutional Review Board.

**Table 1:** Physical Fitness Characteristics of Participants.

	Height (inches)	Weight (lbs)	*VO*_2_ peak(ml/min./Kg)	2 mile time(secs)	Army PhysicalFitness Test Score
mean(SD)	70.34 (2.8)	183.42 (22.4)	50.36 (5.1)	811.6 (46.3)	272.4 (13.6)
N	10	10	10	8	9

### Materials

#### Clothing and Load Components

Volunteers were tested in an unloaded (no load) configuration and a loaded configuration while outfitted with a rifleman’s approach march load. The unloaded configuration consisted of basic clothing (combat boots, socks, T-shirt, and shorts). A simulated M4 carbine was carried in both hands in front of the body (i.e., in the “ready position”) during periods of treadmill walking. The M4 carbine had a response device affixed to the barrel of the weapon to record responses for the cognitive tasks. The weight of the items comprising the no load condition, including the M4, totaled 6.2 kg. The approach march load included all components of the no load condition plus: Improved Outer Tactical Vest (IOTV) with four small arms protective inserts (SAPI) plates; the Tactical Assault Panel (TAP) with pouches loaded with Soldier items; and the medium assault pack of the Modular Lightweight Load-carrying Equipment II (MOLLE II) system. The components comprising the approach load are standard-issue Army items. Soldier items were placed in the pack to obtain a pack center of mass (COM) that is high and close to the wearer’s back. The total weight for the approach load was 40 kg. This load was, on average, 48.6% of the participant’s body weight (*SD* = 5.6%, range = 38.7–56.8%).

#### Go No/Go Auditory Task Stimuli

M4 and AK47 gun fire sounds were used as the stimuli for the auditory task. The duration of both sounds was 500 ms and the volume of both files was normalized.

#### Visual Target Detection Task

A static scene depicting an urban Middle Eastern environment was presented across three 80” televisions positioned approximately 15 feet from the treadmill (approximating 60° horizontal and 12° vertical field of view). The 60 targets were local nationals placed within the scene. Only locations that were physically possible were used (e.g., no targets were placed in the sky). No target appeared in the same location as any other target.

### Procedure

#### Treadmill Walking

Participants attended two orientation sessions to familiarize them with walking on the treadmill and the cognitive tasks. During these sessions *VO*_*2* Peak_ and body dimension measurements were collected as well. After completing the orientation session participants came in for a total four visits with two rest days in between. On four separate test days, participants walked for 120 minutes on the treadmill at a speed of 4.8 kph. Participants took part in each load/grade condition in a repeated-measures design: (1) Approach Load-Constant Grade: carrying an approach march load (40 kg), and walking at a +4% grade for 60 minutes followed by an additional 60 minutes at a constant -8% grade; (2) Approach Load-Varied Grades: carrying an approach march load (40 kg), and walking at a +4% grade for 60 minutes followed by an additional 60 minutes at variable grade (changing between -8%, 0, +4% every 10 minutes); (3) No Load-Constant Grade: without a load, walk at a +4% grade for 60 minutes followed by an additional 60 minutes at a constant -8% grade; and (4) No Load-Varied Grades: without a load, walk at a +4% grade for 60 minutes followed by an additional 60 minutes at variable grade (changing between -8%, 0, +4% every 10 minutes in that order). The order in which participants took part in each condition was counterbalanced across participants in a partial Latin square.

#### VO_2Peak_ Measurements

The determination of ${\dot{V}\text{O}}_{2\text{Peak}}$ was completed during the orientation sessions on a separate day prior to beginning the study. Peak oxygen uptake was measured using a continuous, uphill, stepwise, treadmill protocol. Participants first warmed up on the treadmill for 5 minutes at 1.12 m/s (walk) followed by an additional 5 minute warm up at 2.24 m/s (jog) on a level grade. The participants then began running on the treadmill at 2.68 m/s on a 5% grade. The COSMED Quark CPET metabolic cart was used to monitor oxygen uptake using an oronasal mask and flexible hose. Every two minutes, the treadmill grade was increased by 2.5%, without changing the treadmill speed. The participants was considered to be at peak oxygen uptake if, one minute after an increase in incline, oxygen uptake has not increased by at least 2 ml/kg/min.

#### Percentage of VO_2Peak_ Measurements

During each test session, participants donned the COSMED oronasal mask every ten minutes during the prolonged march to measure breath-by-breath oxygen consumption. Oxygen consumption data was collected for two minutes during steady state walking and the data was averaged over each two minute interval. This allowed for a measure of percentage VO_2Peak_ during various time points of the two hour bout of walking. Measurements were taken at 10, 20, 30, 40, 50, 60, 70, 80, 90, 100, and 110 minutes of walking, in between performance of the cognitive tasks.

#### Auditory Go No/Go Task

Participants performed the auditory go no/go task starting at 5 minutes into walking and continued to perform the task every 20 minutes up until 110 minutes for a total of six 5 minutes blocks of auditory go no/go task performance. During this task participants were presented with either M4 or AK47 recorded gun fire sounds for 500 ms with a variable inter-stimulus interval of 1500–1800 ms. During this task, participants performed two blocks for 5 minutes total with one block consisting of 80% go trials (68 trials; AK47 gun fire) and 20% no-go trials (17 trials, M4 gun fire), for a total of 85 trials and a second block consisting of only go trials (total of 45 trials). Participants were instructed to press the button on the response device affixed to their weapon as quickly as possible when hearing AK47 fire, but to withhold from responding when hearing M4 fire. The M4/AK47 sounds were chosen to emulate a common friend/foe (respectively) determination used during soldier operations. During the second block participants were informed that there would be no no-go trials in order to determine uninhibited reaction time.

#### Visual Target Detection Task

**Fig 1:**
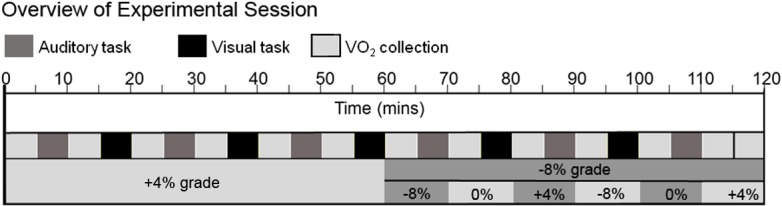
Overview of experimental session with timing of data collections (in minutes), and changes in treadmill grade.

Starting at 15 minutes into walking, participants performed the visual target detection task every 20 minutes up until 100 minutes into walking for a total of five 5 minutes blocks of the visual target detection task. In between both cognitive tasks, percent of *VO*_*2Peak*_ was measured. See Fig 1 for an overview of the experimental session.

### Testing Equipment

For prolonged treadmill walking, participants walked on an integrated force plate treadmill, fabricated by AMTI (Watertown, MA). The treadmill is comprised of two synchronized treadmills side by side on a single platform. The gap between the belts is less than 1.0 cm. The treadmill motors are linked and synchronized such that, as the speed of one treadmill belt changes, the speed of the other belt automatically follows. The treadmill can attain speeds of up to 18.03 kph and can be set at grades of +/- 25%. The treadmill and the software that controls it were designed to enable changes in speed and grade to be made while an individual is walking. The force plates instrumented in the treadmill measure ground reaction forces in three planes. Data are sent from the force transducers in the treadmill to a dedicated computer, which also receives information about the treadmill speed and incline. The COSMED Quark CPET metabolic cart was used to monitor oxygen uptake using an oronasal mask and flexible hose.

### Data Analysis

#### Percentage of VO_2Peak_

Percentage of VO_2peak_ was calculated for each participant in 10 minute intervals referenced to the participant’s VO_2peak_ measured during the orientation session. For the purposes of this study, the onset of physical fatigue was defined as when participants reached 50% of their VO_2peak._ Because the percentage of body weight for the load carried varied slightly for participants, we examined if percentage of body weight significantly influenced exertion levels (as measured by percentage of VO_2peak_) by correlating percentage of body weight with percentage of VO_2peak_ for each of the load conditions. We found no relationship between percentage of VO_2peak_ and percentage of body weight for the load carried (all *r’s* < .29, *p’s* > .4). To determine if participants were significantly more physically fatigued in the loaded compared to loaded conditions, we performed a repeated measures ANOVA for the first hour (i.e., constant grade) of walking with the variables of load (unloaded/loaded) and time (10, 20, 30, 40, 50, 60 minutes). To analyze the second hour of walking, we included the additional variable of grade, as starting at 65 minutes the grade of treadmill was either a constant downhill grade, or changed in grade every 10 minutes. For time, we analyzed the following time points: 70, 80, 90, 100, 110 minutes. The VO_2_ differences were used to confirm that participants were more fatigued in the loaded compared to unloaded condition. Due to the small sample size, VO_2_ measures were only entered as covariates in the ANOVAs with the cognitive performance. See below for further explanation analysis of the cognitive performance data.

#### Analysis of Cognitive Performance

While we analyzed the first and second hours separately for the percentage of VO_2peak_ because the grade changes during the second hour were expected to drive a different pattern of VO_2peak_ measures than in the first hour, we were interested in changes in cognitive performance over the entire two hour walking bout. Therefore, we performed two analyses: one examining the effect of load and exertion on go no/go and visual target detection across the whole two hours, and another analysis examining the effect of grade and load condition during the second hour separately.

#### Auditory Go No/Go Task

Within-subjects repeated measures ANOVAs were performed with the independent variables of time block (5, 25, 45, 65, 85, 105 minutes), load (no load, 40 kg load), and grade (second hour variable, downhill). We conducted a trend analysis by testing for linear, quadratic, and cubic curve estimates via polynomial contrasts. In the case of a significant interaction, we conduct the same ANOVA and contrasts within conditions of interest to test for linear, quadratic, and cubic trends. The dependent variables were sensitivity (*d’*), calculated using the following formula: *d’* = z(hits)–z(false alarms), criterion (c) calculated as c = -.5*(z(hits)+z(false alarms)), the proportion of false alarms, the proportion of hits, reaction time for go trials in a go no/go block vs. reaction time for go trials in a go only block (additional variable of RT type), and reaction time for a go after a no-go. For criterion, note that negative values indicate a bias towards responding yes. Because some participants made no false alarms during some time blocks and load conditions, we were not able to calculate reaction times for no-go trials.

To examine the effect of grade during the second hour, these dependent measures were entered into a repeated measures ANOVA with the independent variables of time block (65, 85, 105 minutes–the blocks in the second hour), load (no load, 40 kg load), and grade (variable, downhill). For post-hoc comparisons, the Bonferroni correction was applied. An ANCOVA with the same factors as above was performed with the covariate of mean centered VO_*2Peak*_, however none of the ANCOVAs found a different pattern of results or interactions with VO_*2Peak*_ after removing one outlier (all *F’s* < .98, all *p’s* > .41), with the exception of reaction time during the all go trial block. Therefore, the results presented do not include the covariate analysis with the exception of the one analysis with reaction time for all go trial block.

#### Visual Target Detection Task

Within-subjects repeated measures ANOVAs were performed with the independent variables of time block (15, 35, 55, 75, 95 minutes), load (no load, 40 kg load), and grade (second hour variable, downhill). We conducted a trend analysis by testing for linear and quadratic curve estimates via polynomial contrasts. In the case of a significant interaction, we conduct the same ANOVA and contrasts within conditions of interest to test for linear, quadratic, and cubic trends. The dependent variables were reaction time and accuracy for target trials. For the accuracy measure a perfect accuracy score equaled 30. In addition, to examine the effect of grade during the second hour, these dependent measures were entered into a repeated measures ANOVA with the independent variables of time block (75 and 95 minutes–the blocks in the second hour), load (no load, 40 kg load), and grade (variable, downhill). For post-hoc comparisons, the Bonferroni correction was applied. An ANCOVA with the same factors as above was performed with the covariate of mean centered VO_*2Peak*_, however none of the ANCOVAS found a different pattern of results or interactions with VO_*2Peak*_ therefore the result presented do not include the covariate analysis (all *F’s* < .65, all *p’s* > .5).

## Results

Results are presented below examining the effects of load, grade, and time walking on percentage of VO_2Peak_ for the first and second hours of walking separately. Performance on the auditory go no/go task and the visual target detection task was examined across the two hours and for the second hour separately.

### Percentage of VO_2Peak_

During the first hour of walking, participants had higher percentage of VO_2Peak_ in the loaded compared to unloaded condition, *F*(1,7) = 90.84, *p* < .001, *ƞ*_*p*_^*2*^ = .91. There was also a significant main effect of time, *F*(5,35) = 5.4, *p* = .001, *ƞ*_*p*_^*2*^ = .38, with corrected pairwise comparisons revealing significant differences between the measurement taken at 10 and 40 minutes (*p* = .001) and 60 minutes (*p* = .003). The interaction between load condition and time was not significant, *F*(5,35) = .94, *p* = .37, *ƞ*_*p*_^*2*^ = .12.

**Fig 2:**
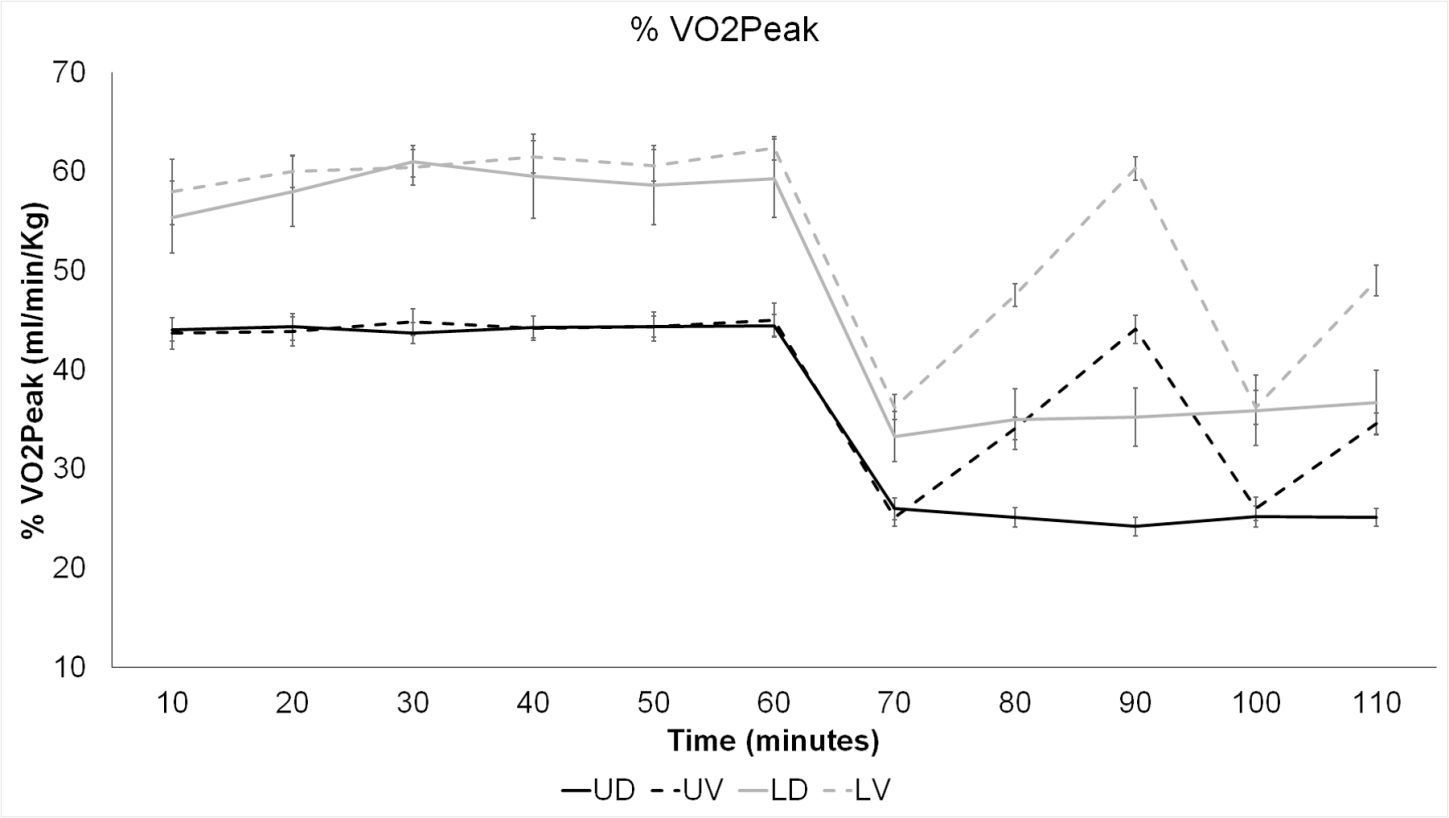
Means and standard errors of percentage of VO_2Peak_ by time, for each of the two load (loaded and unloaded) and grade (downhill, variable) conditions. UD: unloaded, downhill; UV: unloaded, variable; LD: loaded, downhill; LV: loaded, variable.

During the second hour, when grade was manipulated, participants in the loaded condition had a higher percentage of VO_2Peak_ than in the unloaded condition, *F*(1,9) = 57.4, *p* < .001, *ƞ*_*p*_^*2*^ = .86. There was also an interaction between load, grade, and time, *F*(4,36) = 6.64, *p* < .001, *ƞ*_*p*_^*2*^ = .43. Follow-up comparisons examining the loaded and unloaded conditions separately revealed lower percentages of VO_2Peak_ in the downhill condition compared to the variable condition for both load conditions, loaded: *F*(1,9) = 20.76, *p* = .001, *ƞ*_*p*_^*2*^ = .7; unloaded: *F*(1,9) = 60.61, *p* < .001, *ƞ*_*p*_^*2*^ = .88. In addition, for both load conditions, grade also interacted with time reflecting higher percentage of VO_2Peak_ in the variable condition when walking on a flat or uphill incline (*p’s* < .001), but not differing when walking downhill (*p’s* > .17), see Fig 2_._

### Auditory Go No/Go Task

Results are presented for overall performance across the two hour session; however an analysis was performed looking at the second hour of walking only, during which the grade participants walked at either changed to downhill (-8%) or a variable grade (change between downhill, flat, or uphill every 10 minutes). The analysis of the second hour separately did not show any interactions between grade and load, only main effects of grade that parallel the findings in the analysis of the two hours overall, therefore, only results from the overall analysis are reported below.

#### Sensitivity (*d’*)

**Fig 3:**
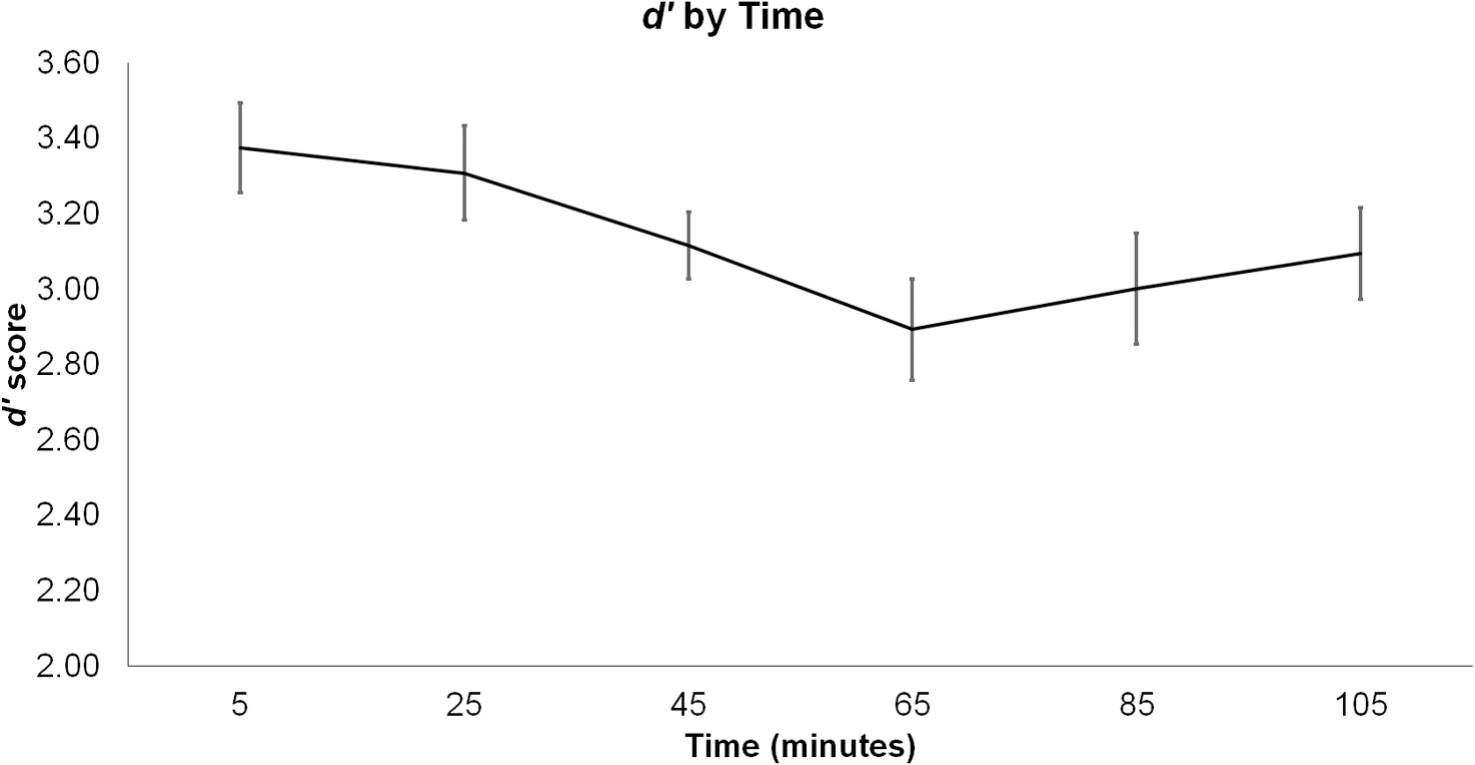
Means and Standard Errors of *d*’ scores over time collapsed across both load conditions (note: higher *d’* scores = better performance).

Participants had lower *d’* scores in the loaded compared to unloaded condition, as indicated by a linear main effect of load, *F*(1,9) = 9.49, *p* = .013, *ƞ*_*p*_^*2*^ = .513; *M*_*loaded*_ = 2.82, *SD*_*loaded*_ = .52; *M*_*unloaded*_ = 3.44, *SD*_*unloaded*_ = .38. Regardless of load condition, performance decreased across the six time blocks, as indicated by a linear main effect of block, *F*(5,45) = 5.65, *p* < .001, *ƞp* ^*2*^ = .386. Bonferroni corrected (critical *p* < .0033) pairwise comparisons revealed this main effect was driven by better performance at 25 minutes, *M* = 3.31, *SD* = .40, compared to at 65 minutes, *M* = 2.89, *SD* = .43, *p* = .001, see Fig 3. None of the other pairwise comparisons reached significance (all *p*’s > .0033). In addition, load and time block did not interact (*F* < 1.7, *p* > .17).

#### Criterion (*c*)

In addition to having less sensitive responses, there was also a shift in the response criterion when participants were in the loaded versus unloaded condition. Specifically, participants in the loaded condition were biased towards responding yes more so than in the unloaded condition, linear main effect of load, *F*(1,9) = 6.34, *p* = .033, *ƞ*_*p*_^*2*^ = .413; *M*_*loaded*_ = -.416, *SD*_*loaded*_ = .25; *M*_*unloaded*_ = -.347, *SD*_*unloaded*_ = .20. No other main effects or interactions reached significance (all *F’s* < 1.85, all *p’s* > .12).

#### Proportion of False Alarms and Proportion of Hits

**Fig 4:**
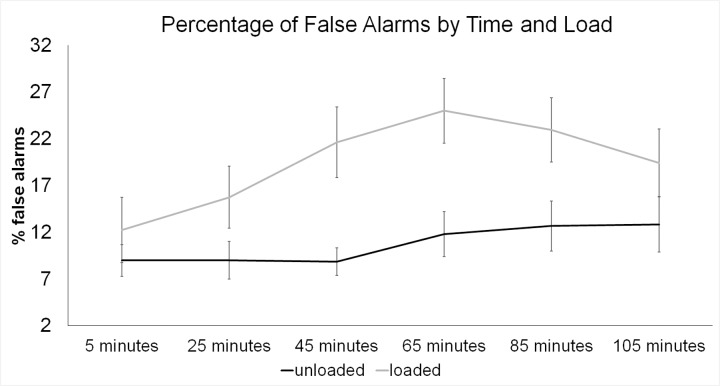
Means and standard errors of percentage of false alarms over time and by load condition (note: higher % false alarms = worse performance).

The analysis examining proportion of hits only found a linear trend towards better performance in the unloaded condition compared to the loaded condition, *F*(1,9) = 5.06, *p* = .051, *ƞ*_*p*_^*2*^ = .36. Therefore, it appears the effect observed on *d’* scores was driven by the proportion of false alarms rather than the hit rate, as there was a significantly higher proportion of false alarms in the loaded compared to unloaded condition, linear main effect of load, *F*(1,9) = 14.48, *p* = .004, *ƞ*_*p*_^*2*^ = .617, *M*_*loaded*_ = .19, *SD*_*loaded*_ = .1; *M*_*unloaded*_ = .097, *SD*_*unloaded*_ = .07, and the proportion of false alarms differed between time blocks, linear main effect of block, *F*(5,45) = 5.32, *p* = .001, *ƞ*_*p*_^*2*^ = .371. Corrected comparisons (critical *p* = .0033) revealed overall worse performance 65 minutes into walking compared to performance after 5 minutes of walking, *p* = .002, *M*_*5mins*_ = .1, *SD*_*5mins*_ = .08; *M*_*65mins*_ = .18, *SD*_*65mins*_ = .09. None of the other corrected comparisons reached significance, all other *p*’s > .006. In addition to these main effects, there was a quadratic interaction between load condition and time block, *F*(5,45) = 2.56, *p* = .041, *ƞ*_*p*_^*2*^ = .22. Corrected pairwise comparisons (critical *p* < .0083) between the loaded and unloaded conditions for each of the six time blocks revealed a higher proportion of false alarms in the loaded condition compared to the unloaded condition at 45 minutes, *t*(9) = 3.94, *p* = .003, *d* = 1.38, at 65 minutes, *t*(9) = 4.64, *p* = .001, *d* = 1.35, and a marginal effect at 85 minutes, *t*(9) = 3.29, *p* = .009, *d* = 1.04. See Fig 4. In addition, to explore the quadratic element of the interaction between load and time block, each load condition by time block was examined separately with a repeated measures ANOVA. For the loaded condition, there were linear, *F*(1,9) = 10.57, *p* = .010, *ƞ*_*p*_^*2*^ = .54, and quadratic effects of time, *F*(1,9) = 5.13, *p* = .05, *ƞ*_*p*_^*2*^ = .36, whereas for the unloaded condition, there was only a linear trend for time block, *F*(1,9) = 3.88, *p* = .080, *ƞ*_*p*_^*2*^ = .17. Overall performance was better in the unloaded condition, however, it did decrease over time in a linear fashion. In the loaded condition, performance decreased over time, but as reflected by the quadratic trend, false alarm rates decreased towards the end the testing period (see Fig 4).

#### Reaction Time

**Fig 5:**
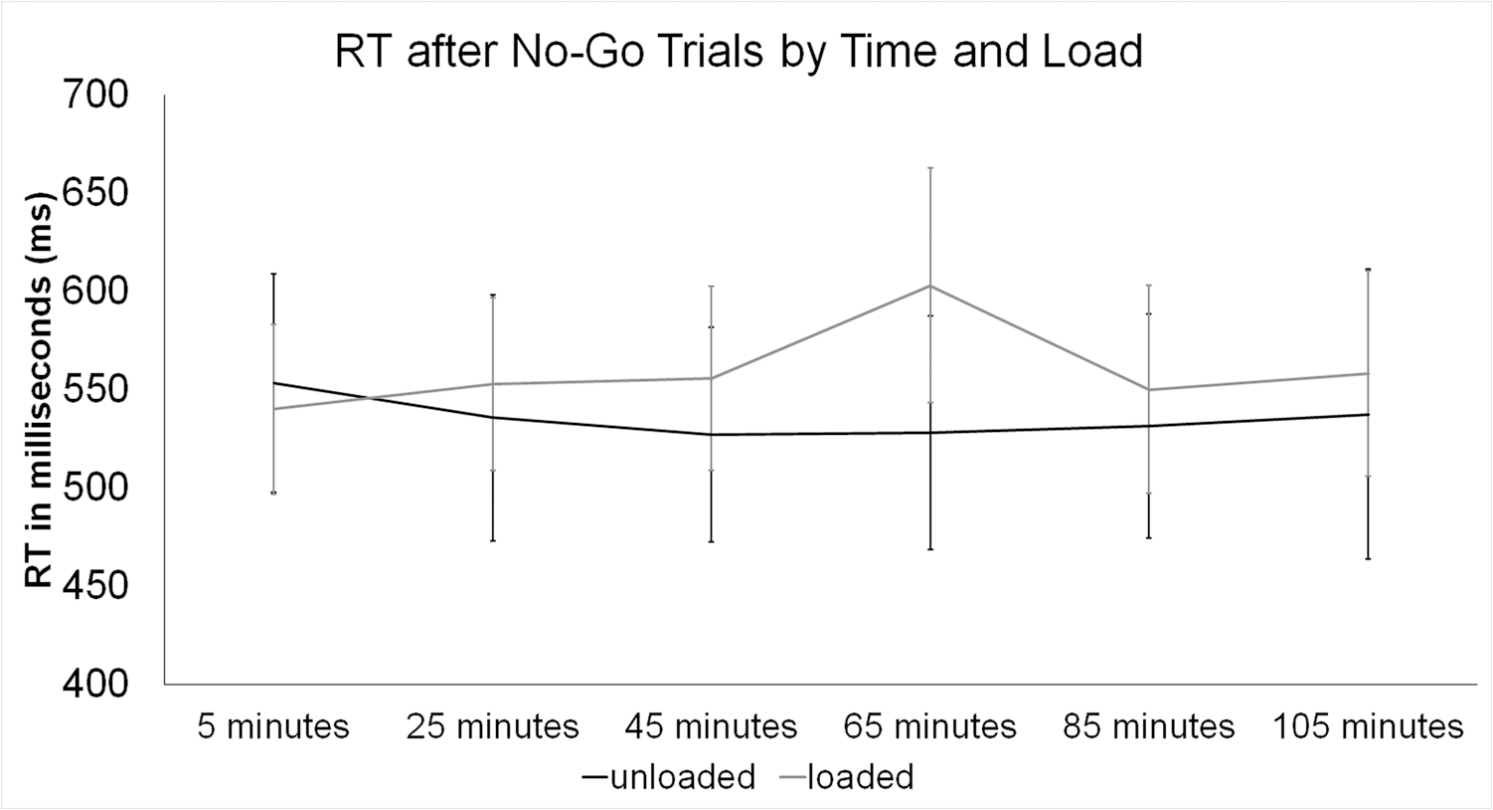
Means and standards errors of reaction times (RTs) for go trials after a no-go trials over time and by load condition.

Comparing reaction times across the two tasks (go trials with no-go trials and all go trials), there was a main effect of trial type, *F*(1,9) = 41.15, *p* < .001, *ƞ*_*p*_^*2*^ = .82, with the reaction time for trials in the all go blocks being faster than trials with go and no-go trials intermixed; *M*_*allgo*_ = 462.86 ms, *SD*_*allgo*_ = 75.29 ms; *M*_*go/no-go*_ = 565.43 ms, *SD*_*go/no-go*_ = 62.06 ms. However, trial type did not interact with load or trial block (all *F*’s < 1, *p*’s > .4). When comparing reaction times for the go/no-go blocks by load condition, there was a interaction between load condition and time block, *F*(5,45) = 3.39, *p* = .011, *ƞ*_*p*_^*2*^ = .27. For this interaction there were no linear, quadratic or cubic elements that reached significance, however, there was a trend towards linear and quadratic elements to this interaction, *p’s* > .054. To examine the above reported interaction, post-hoc corrected comparisons (*p* < .0083) were performed, revealing at 65 minutes of walking, in the loaded condition, participants had significantly longer reaction times than in the unloaded condition (*t*(9) = 3.49, *p* = .007, *d* = .24; *M*_*loaded*_ = 605.27 ms, *SD*_*loaded*_ = 205.63 ms; *M*_*unloaded*_ = 556.18 ms, *SD*_*unloaded*_ = 203.88 ms). Examining the reaction time to a go trial after having a no-go trial, the same pattern was observed with load and time block interacting with quadratic elements, *F*(5,45) = 4.14, *p* = .004, *ƞ*_*p*_^*2*^ = .32. Post-hoc corrected comparisons (*p* < .0083) revealed at 65 minutes of walking, in the loaded condition, participants had significantly longer reaction times than in the unloaded condition (*t*(9) = 4.97, *p* = .001, *d* = .4; *M*_*loaded*_ = 602.83 ms, *SD*_*loaded*_ = 188.88 ms; *M*_*unloaded*_ = 527.98 ms, *SD*_*unloaded*_ = 188.48 ms). The quadratic element of this interaction is reflected by this longer RT at 65 minutes of walking for the loaded condition compared to the unloaded condition (see Fig 5).

The only significant effects in an ANCOVA analysis was found when examining reaction times during the block when participants responded to only go trials. Using the covariate of VO_2Peak_ when comparing the loaded and unloaded conditions, there was a significant interaction between load and VO_2Peak_ with a larger difference in reaction time between the loaded and unloaded condition with lower VO_2Peak_ scores, *F*(1,8) = 70.01, *p* < .001, *ƞ*_*p*_^*2*^ = .90. To confirm this pattern, a correlation between the reaction time difference (loaded–unloaded) and mean centered VO_2Peak_ scores was performed, revealing a negative correlation between VO_2Peak_ scores and the difference in performance in the loaded and unloaded conditions, *r*(9) = .899, *p* = .001.

#### Visual Target Detection Task

Results below are for both overall performance across the two hour session and an analysis examining only the second hour of walking, during which the grade participants walked at either changed to downhill (-8%) or a variable grade (change between downhill, flat, or uphill every 10 minutes). Reaction time and accuracy were analyzed with the factors of time block (1–6), load (no load, 40 kg load), grade (second hour variable, downhill), and target location (left, right, center).

#### Accuracy

Over time, participants’ performance initially decreased, then improved, as demonstrated by a main effect of time block with linear: *F*(1,9) = 15.36, *p* = .004, *ƞ*_*p*_^*2*^ = .631 and quadratic elements, *F*(1,9) = 8.44, *p* = .017, *ƞ*_*p*_^*2*^ = .484, *M*_*15mins*_ = 6.97, *SD*_*15mins*_ = .53, *M*_*35mins*_ = 6.86, *SD*_*35mins*_ = .55, *M*_*55mins*_ = 6.72, *SD*_*55mins*_ = .54, *M*_*75mins*_ = 7.21, *SD*_*75mins*_ = .81, *M*_*95mins*_ = 7.38, *SD*_*95mins*_ = .52. Specifically, accuracy was higher at 95 minutes of walking compared to 15, 35, and 55 minutes into walking (*p’s* < .01). However, there was no interaction with load or grade nor were there main effects of grade or load (all *F’s* < 2.5, *p’s* > .11). There were also no significant linear, quadratic, or cubic trends. Examining performance for the 2^nd^ hour only did not reveal any significant main effects or interactions, all *F’s* < 2.25, all *p’s* > .17.

#### Reaction Time

When examining reaction time for detecting targets, there was a linear main effect of time block, *F*(1,9) = 12.65, *p* = .006, *ƞp* ^*2*^ = .584 with reaction times becoming slower, and significantly slower when comparing performance at 35 and 55 minutes of walking, *p’s* < .017, *M*_*15mins*_ = 837.35 ms, *SD*_*15mins*_ = 61.52 ms, *M*_*35mins*_ = 843.8 ms, *SD*_*25mins*_ = 59.28 ms, *M*_*55mins*_ = 887.68 ms, *SD*_*55mins*_ = 70.1 ms, *M*_*75mins*_ = 880.66 ms, *SD*_*75mins*_ = 87.69 ms, *M*_*95mins*_ = 892.82 ms, *SD*_*95mins*_ = 76.41 ms. The linear main effect of load condition did not reach significance, *F*(1,9) = 4.61, *p* = .060, *ƞp* ^*2*^ = .34, although it approached significance with reaction times being slower in the loaded (*M*_*loaded*_ = 884.59 ms, *SD*_*loaded*_ = 72.21 ms) compared to unloaded condition (*M*_*unloaded*_ = 852.34 ms, *SD*_*unloaded*_ = 61.77 ms). All other main effects and interactions did not reach significance (all *F’s* < 3.2, all *p*’s > .11) and there were no other significant trends observed.

Examining reaction times during the second hour of walking, when treadmill grade was manipulated to be either constantly downhill or changing every 10 minutes between flat, uphill, and downhill, there was a linear main effect of load condition, *F*(1,9) = 14.82, *p* = .004, *ƞ*_*p*_^*2*^ = .622. Reaction times were longer in the loaded (*M*_*loaded*_ = 907.76 ms, *SD*_*loaded*_ = 76.5 ms) compared to unloaded condition (*M*_*unloaded*_ = 865.73 ms, *SD*_*unloaded*_ = 80.1 ms).

## Discussion

In the current study, participants walked for two hours while either carrying no load or a 40 kg load and treadmill grade was manipulated during the second hour to be a constant downhill grade or changing every 10 minutes between flat, uphill, and downhill grades. Analysis of the percentage of VO_2peak_ confirmed that participants were significantly more physical fatigued in the loaded condition compared to the unloaded condition and this interacted with the grade change during the second hour. The main cognitive task findings were that performance decreased over time in the loaded condition relative to the unloaded condition on the go no/go auditory task, as measured by the proportion of false alarms. Specifically, during minutes 45 and 65, participants’ performance was significantly worse in the loaded compared to unloaded condition. This parallels the peak in percentage VO_2peak_, indicating participants in the loaded condition were likely more physically fatigued at these time points. There were also shifts in response criterion (as measured by *c*) and sensitivity (as measured by *d’)* in the loaded condition compared to the unloaded condition with participants in the loaded condition being more likely to shift their criterion towards responding yes and not surprisingly also having lower *d’* scores and increased false alarm rates. As expected, participants showed a pattern of slowed responding in the go/no-go block compared to all go block, with longer reaction times for the go/no-no block. This did not interact with load condition, however, reaction times overall were longer during the second hour of the prolonged march for the loaded condition compared to the unloaded condition. In contrast, physical fatigue (effects over time) and carrying a 40 kg load appeared to have little effect on the visual target detection task. Only during the separate analysis of the second hour only did we find that reaction times were slower for the loaded condition compared to the unloaded condition.

It appears that performance on the go/no-go task is more sensitive to the effects of prolonged, acute exercise, than the visual target detection task, although the null findings for the visual target detection should be interpreted with caution due to the small sample size. However, the data were analyzed with three additional participants (total N = 13) who had data for the visual target detection task, but not the go/no-go task and the same pattern of null effects was also observed. The results are in line with the idea that higher level, frontally mediated cognition is affected by exertion and exercise more so than lower level cognitive processes during exercise as suggested by the transient hypofrontality hypothesis [18]. However, some of our findings are inconsistent with this hypothesis, namely that we found slowed reaction times during the second hour of the visual target detection task. In addition, we found that reaction times in the go/no-go task were slower in the loaded condition. While the hypofrontality hypothesis would suggest performance relying on sensorimotor pathways should remain intact, our study and several other studies have found evidence against this hypothesis by demonstrating decrements in performance for simple psychomotor tasks, for example see [13]. The differing findings in this study and others, suggests the relationship between acute exercise, higher level cognition, and lower level cognition is more complex than a simple load- or time-mediated effect.

In addition to the transient hypofrontality hypothesis, other factors may play a role in performance decrements during load carriage, including dual-task performance and balance. For instance, auditory monitory performance decreased after a 20 km road march a carrying 34 kg pack, though thought not after carrying 48- and 61-kg packs, perhaps due to increased speed and energy expenditure during this march [21]. In addition, participants carrying a 40 kg load and walking over obstacles impaired vigilance performance [22], suggesting the dual-task of going over obstacles and balancing a load may impact cognitive performance. Further evidence for the role of balancing a load and performing a cognitive task come from a study where participants carried 30% of their body weight had reduced task switching performance, along with balance control [23]. Future should better determine how trade-offs between load carriage, speed and balance influence executive function.

We found no relationship between percentage of VO_2Peak_ and cognitive performance, although a more heterogeneous sample would be desirable in order to examine this relationship in addition to the relationship between VO_2Peak_ and cognitive performance during exercise. Even though we were not able to relate percentage of VO_2Peak_ and cognitive performance, the percentage of VO_2Peak_ measurements confirm that participants were sufficiently fatigued. As can be seen in Fig 1, during the end of the first hour, participants have significantly higher percentage of VO_2Peak_ in the loaded condition compared to the unloaded condition.

The pattern of percentage of VO_2Peak_ indicated there were physiological differences between the grade conditions during the second hours of walking. Physiologically, participants recovered, that is they were at a lower percentage of VO_2Peak_ when walking downhill compared to when the grade was variable during the second hour. However, cognitive performance did not parallel the pattern of VO_2Peak_ measures. We did observe a recovery in performance, measured by percentage of false alarms, in the loaded condition, however, we did not observe any interactions between load condition, time, and the manipulation of grade during the second hour. Therefore, it appears, while participants may have been showing some recovery physically, their cognitive performance was still impacted by the overall level of fatigue they were experiencing during load carriage compared to the no load carriage conditions.

The current study has advantages compared to previous studies, in that the exercise duration was quite long allowing us to measure a longer time course of cognitive performance during exercise; and secondly, we measured performance during exercise. The majority of other studies only have participants exercise for a short duration (< 1 hr) and measure performance pre and post exercise, although some have exceeded the 1 hour threshold [13, 14, 20]. The fact that decrements in performance are not found when comparing pre- vs. post-performance is in support of the idea that hypofrontality is indeed transient and that exercisers recover from this state very quickly after exercise has ceased. One limitation of this study is the small number of participants who were included in the analysis. This could be one reason for the mainly non-significant findings for the visual target detection task.

Overall, the findings of this study suggest that cognitive performance can successfully be measured during acute exercise and appears to be more sensitive to decrements in cognitive performance that result from fatigue during prolonged exercise when comparing load carriage to no load carriage. Specifically, we found a task that taps executive control and response inhibition to be affected by long duration exercise and load carriage, whereas the effects on a visual vigilance task were minimal. These results provide important information for future load carriage requirements for US Army personnel.
